# Rationale, Study Design, and Cohort Characteristics for the Markers for Environmental Exposures (MEE) Study

**DOI:** 10.3390/ijerph17051774

**Published:** 2020-03-09

**Authors:** Rachel McFarland Lucia, Wei-Lin Huang, Andrea Alvarez, Daphne Thampy, Melodie Elyasian, Amanda Hidajat, Kailynn Yang, Danielle Forman, Asana Pebdani, Irene Masunaka, Susie Brain, Diane Heditsian, Vivian Lee, Deborah Goodman, Trina M. Norden-Krichmar, Andrew O. Odegaard, Argyrios Ziogas, Hannah Lui Park

**Affiliations:** 1 Department of Epidemiology, University of California, Irvine, CA 92697, USA; mcfarlar@uci.edu (R.M.L.); weilih2@uci.edu (W.-L.H.); andreaca@hs.uci.edu (A.A.); dthampy@uci.edu (D.T.); melyasia@uci.edu (M.E.); ahidajat@uci.edu (A.H.); kailynny@uci.edu (K.Y.); dforman1@uci.edu (D.F.); apebdani3@gmail.com (A.P.); ikmasuna@uci.edu (I.M.); goodmand@uci.edu (D.G.); tnordenk@uci.edu (T.M.N.-K.); aodegaar@uci.edu (A.O.O.); 2 Patient and Research Advocate, Markers for Environmental Exposures Study, Irvine, CA 92697, USA; susie_brain@yahoo.com (S.B.); diane@declarity.com (D.H.); vlee@aquapartners.net (V.L.); 3 Department of Medicine, University of California, Irvine, CA 92697, USA; aziogas@uci.edu

**Keywords:** environmental exposures, biomarker, DNA methylation, epigenetics, biorepository

## Abstract

Environmental factors have been linked to many diseases and health conditions, but reliable assessment of environmental exposures is challenging. Developing biomarkers of environmental exposures, rather than relying on self-report, will improve our ability to assess the association of such exposures with disease. Epigenetic markers, most notably DNA methylation, have been identified for some environmental exposures, but identification of markers for additional exposures is still needed. The rationale behind the Markers for Environmental Exposures (MEE) Study was to (1) identify biomarkers, especially epigenetic markers, of environmental exposures, such as pesticides, air/food/water contaminants, and industrial chemicals that are commonly encountered in the general population; and (2) support the study of potential relationships between environmental exposures and health and health-related factors. The MEE Study is a cross-sectional study with potential for record linkage and follow-up. The well-characterized cohort of 400 postmenopausal women has generated a repository of biospecimens, including blood, urine, and saliva samples. Paired data include an environmental exposures questionnaire, a breast health questionnaire, dietary recalls, and a food frequency questionnaire. This work describes the rationale, study design, and cohort characteristics of the MEE Study. In addition to our primary research goals, we hope that the data and biorepository generated by this study will serve as a resource for future studies and collaboration.

## 1. Introduction

Studies suggest that human cancers are due to a combination of factors, including heredity, random mutations, and environmental factors [1,2,3,4,5]. In addition, environmental factors, including exposure to tobacco smoke, alcohol consumption, obesity, inadequate physical activity, radiation, and chemical exposures, have been shown to be associated with many other major chronic diseases, including cardiovascular disease [6], stroke [7], chronic obstructive pulmonary disease [8], and diabetes [9] as well as other conditions ranging from infertility to neurodegenerative diseases [10,11]. While some of these factors are well-established risk factors for disease, the evidence for many others is insufficient.

Reliable estimates of exposure are needed to analyze the potential relationships between exposures and health. Self-reporting is commonly used to assess environmental exposures in epidemiological research. However, compared to direct measurements, such as biomarker measurement, self-reported data are prone to recall bias, conscious or unconscious. For example, studies have found that about 20% of pregnant women with cotinine levels indicative of current smoking failed to report their smoking habits when asked [12,13]. For many other environmental exposures, self-report is difficult because individuals may be unaware of the dose, frequency, or time frame of exposure. Biomarkers can serve as objective markers for environmental exposures which may be used to improve our understanding of how exposures relate to health.

In recent years, interest has mounted in identifying epigenetic markers for exposures, or markers based on differences in non-sequence modifications of the DNA [14,15,16]. DNA methylation is the best-studied epigenetic mechanism in this area and has several properties that make it ideal for marker development. First, it has a desirable balance between plasticity (allowing a biologic response to environmental insults) and long-term stability (allowing the assessment of past exposures). This has allowed the identification of DNA methylation-based biomarkers of numerous environmental exposures, most notably cigarette smoking, which has been consistently associated with methylation at sites within the *AHRR* and *F2RL3* genes in numerous studies [17,18,19]. In one study, *AHRR* methylation predicted current smoking status with an area under the curve (AUC) of 0.99 [20]. Studies of smoking have also demonstrated the long-term stability of DNA methylation biomarkers, with one study showing that former smokers exhibit an intermediate methylation status and may return to a “nonsmoking” state over a period of at least 10 years [21]. Another useful quality is that DNA methylation can be relatively easily measured from blood, saliva, or tissue DNA, including banked DNA, which will allow leveraging of existing large cohort studies and biorepositories in order to identify associations of environmental exposures with health outcomes. Specific patterns of DNA methylation have been identified for some other environmental exposures, including air pollution [22,23] and polycyclic aromatic hydrocarbons [24,25], but the impact of many environmental exposures on the epigenome remains unknown.

**Figure 1:**
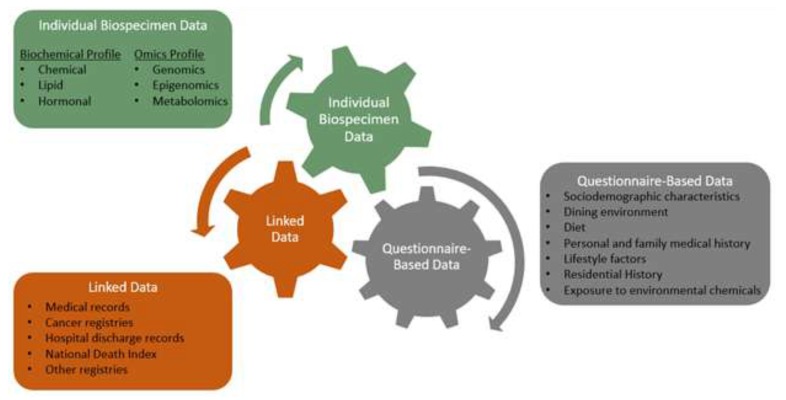
Conceptual model for the MEE Study. The conceptual model includes both completed and potential (future) data collection. At the time of this writing, completed collections include all listed questionnaire-based data, biospecimens, epigenomic profile, urinary levels of glyphosate, organophosphate pesticides, and bisphenols A and S, and mammogram reports.

This paper describes the design, methods, and participants of the Markers for Environmental Exposures (MEE) Study, a cross-sectional study conducted from 2017–2019 comprised of 400 postmenopausal women aged 45 to 66 years in Southern California. Questionnaire data, medical record data, and biospecimens were collected from each participant. The repository of data and biospecimens (and molecular profiles obtained from them) can be linked to future cancer registry data and other publicly available data (Figure 1). This conceptual framework will support the long-term vision of the MEE Study to (1) identify biomarkers, especially epigenetic markers, of environmental exposures, such as pesticides, air/food/water contaminants, and industrial chemicals that are commonly encountered in the general population; and (2) support the study of potential relationships between environmental exposures and health and health-related factors.

## 2. Materials and Methods

### 2.1. Study Design

The MEE Study is a cross-sectional study with potential for follow-up data collection and linkage. The study was approved by the University of California, Irvine (UCI) Institutional Review Board (HS# 2016-3127), and the investigators worked closely with a panel of three patient and research advocates from the community (S.B., D.H., and V.L.). While the study was originally designed with the intention of identifying epigenetic markers for pesticide exposure and factors related to breast cancer risk, the data and specimens collected can potentially be used in the context of other diseases, conditions, and environmental exposures. All participants provided written consent to participate in the study and HIPAA authorization.

**Figure 2:**
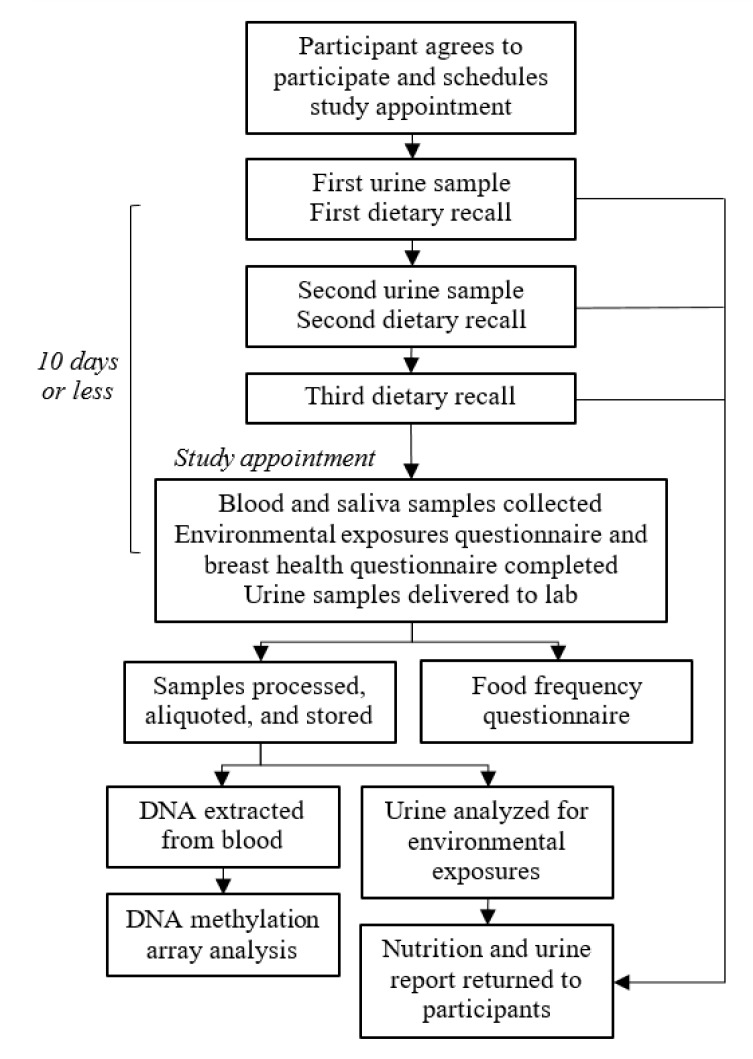
Study workflow. The MEE Study collected biospecimens and questionnaire-based data from 400 postmenopausal women aged 45 to 66 years residing in Southern California.

The study workflow is outlined in Figure 2. Participants were asked to complete six online questionnaires, provide biospecimens, and provide authorization to access their breast health-related medical records (Table 1). The six questionnaires consisted of an environmental exposures questionnaire, a breast health questionnaire, three 24-h dietary recalls completed on three separate days (two weekdays and one weekend day), and a food frequency questionnaire. The biospecimens consisted of two self-collected first-void urine samples, a venous peripheral blood sample, and an optional saliva sample. Study participants received a nutrition report based on their 24-h dietary recalls and a report of their urinary levels of certain pesticides (glyphosate and organophosphates); participants received no monetary compensation.

### 2.2. Study Population

Four hundred (400) participants were recruited from October 2017 to May 2019. Participants were female residents of primarily Orange County, CA. Women were eligible to participate in the study if they were aged 45–66 years, postmenopausal (had not had their period in over one year), and had never been diagnosed with breast cancer or had a mastectomy. Eligibility criteria were based on the study’s main aim of identifying epigenetic markers for pesticide exposure and factors related to breast cancer risk. Age is known to be associated with epigenetic changes [26,27] as well as breast cancer risk [28], and menopause has also been shown to be associated with epigenetic changes [29]. Women with a personal history of breast cancer or mastectomy were excluded in order to capture the population at risk for incident breast cancer. 

**Figure 3:**
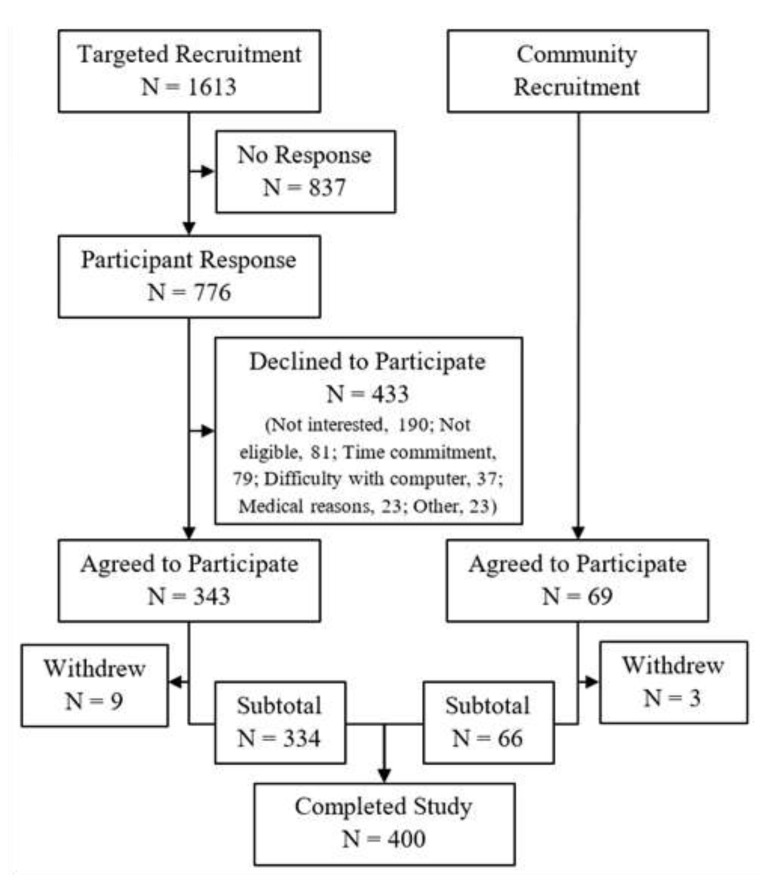
Flow chart of study recruitment. Recruitment for the MEE Study used two primary methods: personalized mailings to members of a breast cancer screening cohort (“Targeted Recruitment”) and general announcements in the community (“Community Recruitment”).

There were two primary methods of study recruitment (Figure 3). The first involved personal mailings to patients who had received a screening mammogram at a University of California, Irvine Health facility and had consented to be contacted for future research studies as part of the Athena Breast Health Network [30]. For women who were eligible based on their most recent breast health questionnaire, three attempts were made to contact them using varied methods (telephone, mail, and email). Participants were also recruited from the broader community through email announcements to UCI employees; announcements/flyers posted on social media, local community boards, and at events; and via word-of-mouth. This method relied on potential participants initiating contact with the study team after seeing the announcement or flyer. Translated materials were available for patients whose primary language was Spanish. The study coordinator was fluent in both English and Spanish.

### 2.3. Specimen and Data Collection

#### 2.3.1. Specimen Collection, Processing, and Storage

After enrolling in the study and scheduling a blood collection appointment, participants were mailed study materials including printed instructions in English or Spanish and two sterile urine collection cups labeled with their study ID. The 4-oz (118 mL) collection cups were individually sealed, made of high-density polyethylene plastic and had a spill-proof, leak-resistant screw-on sealing cap. A security seal on the cap prevented any tampering. Participants were advised that during the 10-day period prior to their scheduled blood collection appointment, they would be contacted on two separate days at 7:30 PM to ask them to self-collect their first-void urine samples the following morning and place the filled collection cups in a freezer (−20 °C) until their appointment. Contact was via text message or email based on participant preference. Participants were not told which days they would be contacted, ensuring capture of their typical behaviors and exposures.

Peripheral blood was collected at the participant’s home or other location of the participant’s choice by the study coordinator, who was also a certified phlebotomist. Three glass Vacutainer^®^ blood collection tubes were used: one containing ethylenediaminetetraacetic acid (EDTA), one containing acid citrate dextrose (ACD), and one with no anticoagulants. Blood tubes were wrapped in an insulated bag and placed in a portable cooler with ice so the samples were cold but did not reach the point of freezing, while the coordinator was in the field. At the time of the blood collection appointment, the study coordinator also collected the previously frozen urine samples and placed them in the portable cooler. Upon arrival at the lab, the urine samples were stored at 4 °C to thaw overnight; the next day, the samples were aliquoted and stored at −80 °C for future analysis. 

Within six hours of blood collection, blood samples were centrifuged at 2000 rpm for 10 min and isolated for serum (from tube with no anticoagulant), plasma (from EDTA and ACD tubes, separately) and buffy coat (from EDTA and ACD tubes, pooled). The serum and plasma were divided into 2 mL aliquots and stored at −80 °C for future use. DNA was extracted from the buffy coat using the QIAamp DNA Blood Maxi Kit (Cat No. 51194, QIAGEN, Hilden, Germany). Extracted DNA was quantitated using the Qubit™ dsDNA HS Assay (Cat No. Q32854, ThermoFisher Scientific, Waltham, MA, USA) and the BioTek Synergy HT (BioTek, Winooski, VT, USA) microplate reader, and stored at −80 °C. Optional saliva samples were collected from the participants in 15 mL Falcon tubes and stored at −80 °C for future analysis.

#### 2.3.2. Environmental Exposures and Breast Health Data Collection

Data on environmental exposures and breast health were collected using electronic questionnaires on the Research Electronic Data Capture (REDCap) and Salesforce platforms. The REDCap electronic data capture tool was hosted at the University of California, Irvine and is a secure, web-based application designed to support data capture for research studies [31]. Salesforce is a secure, cloud-based customer relationship management system which is also used for research questionnaire data collection [32]. Environmental exposure questions included those regarding participants’ residential history, frequency of organic food consumption, frequency of dining out, smoking history, and hormone use history. Organic eating behaviors were assessed with both a general question asking “Do you eat organic food?” as well as food-group specific questions for fruit, vegetables, grains, meat, and dairy, with response choices for each question being seldom or never, sometimes, often or always, and don’t know or not sure. This question is consistent with previous studies of organic eating behaviors [33,34]. Food-group specific questions also included an option for “I do not eat the food” to account for different dietary patterns. Organic food was defined in the questionnaire as food that is either labeled United States Department of Agriculture (USDA) Organic, purchased locally from an organic farm, grown without pesticides in a home garden, or raised on organic feed without hormones and without antibiotics. Participants provided their current residential address, previous cities or ZIP codes, and length of residence at each location. Geocoded addresses can be used to model residential exposure to air pollution, as previously described in similar studies [22,35], or other neighborhood-specific environmental factors, such as green space. Never-smokers were defined as those who had never smoked regularly for 6 months or more. Questions regarding occupation and use of pesticides were added to the questionnaire approximately midway through study enrollment (250 participants were asked these questions). Breast health questions included those regarding the participant’s history of mammography screening, personal and family history of breast and other cancers, personal gynecologic history, alcohol consumption, and physical activity. All questionnaires used branching logic, and the full text of each question for all questionnaires is provided in Table S1. Given the strong association between mammographic density and breast cancer risk [36,37] and its response to exposures such as exogenous hormones [38] and BMI [39], mammographic density (BIRADS categories 1, 2, 3, and 4) was also collected from participants’ most recent mammogram reports.

#### 2.3.3. Dietary Data Collection and Processing

Participants were advised that during the 10-day period prior to their scheduled blood collection appointment, they would be contacted by the study team on three days at 10:00 AM to ask them to complete a 24-h dietary recall for the previous day. Contact was via text message or email according to participant preference. Participants were not told which days they would be contacted, and requests were made to include one weekend day, in order to capture their typical diet. Two recalls were requested on the same days as requests for first-void urine samples so the data would be paired.

Dietary intake data for 24-h recalls were collected and analyzed using the Automated Self-Administered 24-h (ASA24) Dietary Assessment Tool, version 2016, developed by the National Cancer Institute (Bethesda, MD, USA) [40]. ASA24 is modeled on the USDA’s Automated Multiple-Pass Method (AMPM) and collects data using a meal-based approach to evaluate the consumption of specific food groups in addition to overall diet by calculating a dietary score [41] and is available in Spanish. This assessment is able to provide high quality information regarding dietary intake at a low cost with minimal participant burden [42].

Participants were also asked about their usual food consumption, including portion sizes, during the previous 12 months using the Diet History Questionnaire (DHQ II), a freely available food frequency questionnaire (FFQ) developed by the National Cancer Institute [43]. English speakers received the web version of DHQ II and Spanish speakers received a paper version of the DHQ I since a paper version of the DHQ II was not available in Spanish. DHQ I has been validated in three studies [44,45,46]. Updates to the second version included additions of foods and supplements but were minimal such that validation findings are unlikely to be significantly modified. Diet*Calc Software (V1.4.3, National Cancer Institute, Bethesda, MD, USA) [47] was used to analyze the DHQ data and generate nutrient and food group intake estimates. Dietary recalls which had total kcals lower than 500 kcal/day or higher than 3500 kcal/day were removed, which are cutoffs commonly used to exclude outliers in diet data for epidemiologic studies [48].

#### 2.3.4. DNA Methylation Data Collection and Processing

Genomic DNA (1000 ng) samples were bisulfite converted using the Zymo EZ DNA methylation kit (Zymo Research, Irvine, CA, USA) according to the manufacturer’s recommendations at the University of Southern California Molecular Genomics Core Facility. Bisulfite-converted DNA was then used as a substrate for the Illumina Infinium MethylationEPIC BeadChip (Illumina, San Diego, CA, USA), which measures methylation levels at over 850,000 sites throughout the genome [49]. After assessing sample quality using the standard controls included in the array, methylation array data were filtered and normalized using the recommended pre-processing steps for Illumina methylation BeadChip data [50].

#### 2.3.5. Questionnaire Data Curation

Questionnaire data were reviewed and curated, and some variables were regrouped to improve clarity and conciseness. For example, for race/ethnicity, only 5.0% of participants indicated a race/ethnicity other than non-Hispanic white, Hispanic, or Asian. These participants were grouped into the category “Other/Unknown” with those who indicated Unknown or left the field blank. Occupations were classified into six groups based on the Standard Occupational Classification (SOC) from the U.S. Department of Commerce, Office of Federal Statistical Policy and Standards, and O*NET, the U.S. Occupational Information Network [51,52]. Occupation classification was performed by two separate individuals (W.-L.H. and D.F.), and any discrepancies were resolved by a third-party adjudicator (H.L.P.). Participants’ mailing addresses were used to determine their current residences. Cities within Orange County were classified into either North or South Orange County [53]. For physical activity, participants were classified based on whether their weekly physical activity met the Physical Activity Guidelines for Americans, issued by The Department of Health and Human Services, which recommend at least 150 min per week of moderate-intensity aerobic activity, at least 75 min a week of vigorous-intensity aerobic activity, or an equivalent combination of moderate- and vigorous- intensity aerobic activity for adults [54].

#### 2.3.6. Statistical Analysis

Basic cohort characteristics were compared for the two recruitment methods (targeted and community recruitment) using the student’s *t*-test for age and Fisher’s exact test for all other variables. Fisher’s exact test was used rather than the chi-squared test due to small counts in some cells of the contingency table, which may affect the precision of the chi-squared approximation.

## 3. Results

### 3.1. Specimen and Questionnaire Collection

Four hundred postmenopausal women aged 45 to 66 years enrolled in the study (Table 2). All participants provided a blood sample and two urine samples. Over half of the participants (*n* = 223, 55.8%) provided the optional saliva sample. Some urine samples and dietary recalls were not completed on the days requested because participants forgot to complete them, were unexpectedly out of town or unavailable, or due to temporary problems accessing the ASA24 system. In these cases, participants were asked to complete their urine collection or dietary recall the next day that was feasible. Some study appointments had to be rescheduled because participants due to illness or other reasons. The average time between collection of the two urine samples was 2 days (SD 1.7, range 1–17 days). The average time between collection of urine samples and blood samples was 3 days (SD 3.3, range 0–26 days). DNA was extracted from buffy coat of all 400 participants’ blood samples (mean 291.0 ± 140.4 µg DNA extracted, range 10.3–919.8 µg).

All participants completed the breast health questionnaire and environmental exposures questionnaire. After excluding ASA24 dietary recalls with kcals outside of the range of 500 kcals to 3500 kcals, 379 participants (94.8%) had at least one dietary recall, and 67.5% of participants had all three requested recalls (Table 2). Based on the study design, two dietary recalls for each participant were intended to be paired with urine samples. 344 participants (86.0%) had at least one paired dietary recall and 234 participants (58.5%) had both paired dietary recalls. 263 participants (65.8%) completed the food frequency questionnaire. There were no significant differences in questionnaire completion rates between participants from the two recruitment methods (data not shown).

### 3.2. Participant Demographics

Since this study had a specific recruiting age range, the average age of participants was 56.7 years with small variation (SD 4.6 years). The majority of participants were non-Hispanic white (64.3%) followed by Hispanic (18.5%), Asian (10.8%), and Other/Unknown (6.5%). Over 50% of participants graduated from college or had more education. Of the participants who indicated their occupation (N = 250), 81.6% were currently employed either full-time or part-time. 56.4% currently had an occupation related to management, business, science or arts, followed by sales and office-related occupations (15.2%) and service-related occupations (8.0%). Over 90% of participants lived in either North or South Orange County. A small percentage of participants (9.4%) were from Los Angeles, San Bernardino, Riverside and San Diego Counties (Table 2). There were no significant differences between participants from the two recruitment methods for age, race/ethnicity, or education (data not shown).

### 3.3. Lifestyle and Health History

The majority of cohort participants were never-smokers (73.0%) (Table 3). Only 17 participants (4.3%) currently smoked, while 90 participants (22.5%) previously smoked regularly. The majority of cohort participants (68.0%) consumed less than two alcoholic drinks per week or never consume alcohol, while 31.2% reported consuming two or more alcoholic drinks per week. Only 39.0% of participants were meeting physical activity guidelines. The average body mass index (BMI) in the cohort was 26.8 kg/m^2^. 

About 78% of participants had given birth at least once, with a mean age of 27.7 years old at first birth. The average ages of menarche and menopause were 12.8 years and 48.7 years, respectively. 121 participants (30.3%) had either a hysterectomy or oophorectomy, with 62 (15.5%) having had both surgeries. Just over 20% of participants had first-degree relatives who had been diagnosed with breast cancer, while 3.3% had a first-degree family history of ovarian cancer. 60.0% of participants had heterogeneously or extremely dense breasts according to their most recent mammogram. The most commonly reported personal health conditions were back pain (*N* = 84, 21.0%), thyroid disease (*N* = 71, 17.8%), hypertension (*N* = 70, 17.5%), and depression (*N* = 50, 12.5%). Just over 10% (*N* = 44) of participants reported a previous diagnosis of cancer (besides breast cancer). The most commonly reported cancers were non-melanoma skin cancer (*N* = 10), melanoma (*N* = 9), cervical cancer (*N* = 7), and thyroid cancer (*N* = 6). Five participants (1.3%) had previously been diagnosed with ovarian cancer.

### 3.4. Environmental Exposures

Participants were asked questions regarding environmental exposures through their daily activities; responses are summarized in Table 4. Frequency of organic food consumption in the cohort was quite evenly distributed, with 31.8% of participants reporting “often or always” eating organic food and 39.5% reporting “seldom or never” eating organic food. Over 90% of participants primarily drank bottled or filtered water, while 9.0% drank tap water without a filter. Only 13.5% of participants had ever lived on a farm; 5.5% had lived on a farm for more than 10 years. Of the participants who were asked about their use of pesticides, 20.0% (*N* = 50 out of 250) reported using pesticides at home or work within the past week.

## 4. Discussion

Environmental exposures are ubiquitous in daily life and have been associated with a multitude of diseases. However, many environmental exposures are difficult to objectively assess due to inaccurate self-reported data or challenges with biomarker measurement [16]. The development of blood-based biomarkers for environmental exposures will allow us to more accurately study the long-term impact of these exposures by leveraging existing large cohort studies with banked blood and/or DNA samples. We established the MEE Study in order to bridge this gap in knowledge by developing DNA methylation-based biomarkers for environmental exposures and other risk factors for disease. Such biomarkers have been identified for some exposures [18,22,25] but not for many others.

Our targeted approach to recruitment had a high success rate: 20.7% of all potential participants who were contacted and 43.0% of those who responded to our initial contact attempts eventually completed the study. This is likely due to the targeted recruitment pool being members of a breast cancer screening program who consented to being contacted for future research studies. Of the women who were eligible for the study, the most common reasons given for declining to participate were that they were uninterested, had concerns about the time commitment involved, and had concerns about using a computer or mobile phone to complete study questionnaires. Because the community recruitment method relied on participants initiating contact with the study team, it is impossible to know the success rate or reasons for non-participation in this group. However, the general demographics of participants recruited through each method were largely similar (data not shown).

A primary strength of our study is the resulting biorepository of plasma, serum, urine, and saliva as well as extracted DNA from the buffy coat. Plasma and serum can be analyzed for an array of molecules including antibodies and other proteins, antigens, lipids, hormones, and exogenous substances. Multiple urine samples allow for the reasonably representative measurement of chemicals with short half-lives. However, the relatively short time period of biospecimen collection (up to 10 days between urine samples) remains a limitation of the study. Certain exposures of interest are likely to be relatively consistent over time, while others may exhibit strong temporal variability. We plan to leverage the MEE Study population in the future by recruiting a subset of cohort members to a longitudinal study, providing repeated biospecimens which will assess environmental exposures over a longer period of time. While analyses of urinary glyphosate, organophosphates, and bisphenols have been completed, remaining aliquots of stored urine samples are available for analysis of other environmental chemicals as well as metabolomic and electrolyte analysis. Some of the extracted blood DNA has been used for DNA methylation array analysis, and remaining blood DNA can be used for other genetic and epigenetic analyses. Stored saliva samples can also be used for genetic and epigenetic analyses as well as microbiome or biochemical analyses in the future.

Another strength is our comprehensive study data which includes detailed dietary information (multiple 24-h dietary recalls and a food frequency questionnaire), self-reported environmental exposures, lifestyle factors, residential history, occupation, and personal and family health history, especially breast health-related data. While our sample size is much smaller than large cohort studies such as the Women’s Health Initiative or Nurses’ Health Study, this smaller sample size allowed deep, focused characterization of each participant with omics-scale data from diverse biospecimens (blood, saliva, and urine). This data was used to prepare a nutrition and environmental exposure report for return to study participants, and 99% of participants requested to receive the reports. Dietary recalls and first-void urine samples were unannounced, which should provide a more unbiased assessment of usual diet and exposures. Two dietary recalls were paired with urine samples; that is, participants received a request at 7:30 PM one evening to collect their first-void urine the next day, followed by a second message at 10:00 AM to request that they complete a 24-h dietary recall reflecting the previous day’s intake. Participants were not informed at the time of recruitment that urine samples and dietary recalls would be paired, so the request for a urine sample should not have affected the unannounced nature of the dietary recall. In addition, because the urine sample request was sent at 7:30 PM, most of that day’s dietary intake would have already taken place, further reducing the chance of bias. Additionally, participants provided information on their current and former residences, which may be used in the future to assess residential exposure to air pollution as well as other neighborhood factors. However, due to the questionnaire-based assessment of most environmental exposures, in order to minimize participant burden, not all exposures were captured.

A major limitation of the MEE Study is the lack of follow-up and thus health outcome data. The study was originally conceived with a cross-sectional design to allow the development of exposure biomarkers, which does not require health outcome data. This limitation does not affect the primary goal to identify epigenetic markers of environmental exposures, which will support future studies of potential relationships between environmental exposures and health. However, the MEE Study cohort is well-characterized and its participants have shown a high willingness to participate in research. Thus, the data collected can be enhanced in the future through linkage to national and state-level cancer registries, Department of Motor Vehicles and voter registration records, hospital discharge records from the California Office of Statewide Health Planning and Development (OSHPD), mortality records, and other sources of data. Additional data may also be gathered through follow-up questionnaires.

The study cohort was drawn from the primarily suburban area of Orange County, California. Similar to the demographic characteristics of the region, study participants are fairly racially/ethnically diverse with a large proportion being of Asian and Hispanic heritage, and are largely college educated [55]. Generally, the study population exhibited higher levels of healthful behaviors than the general U.S. population. For example, just 4.3% of study participants were current smokers compared to 19.7% of women aged 45–64 nationwide; similarly, 22.3% of study participants had a BMI of 30 kg/m^2^ or greater, compared to 32.4% of women aged 45–64 nationwide [56]. An exception was the adherence to physical activity guidelines: 39.0% of participants met these guidelines compared to 40.9% of women aged 45-64 nationwide [56]. Since Orange County has a large Spanish-speaking population [55], study materials were translated to Spanish; eighteen study participants (4.5%) requested Spanish-language materials and communication. Additionally, the study population is entirely composed of postmenopausal women, which minimizes the biological variance in DNA methylation and other markers due to age, sex, or menopausal status, but may limit the generalizability of the results. Applicability of future study results will need to be validated in other populations. 

## 5. Conclusions

The Markers for Environmental Exposures (MEE) Study is a well-characterized cohort of 400 postmenopausal women residing in Southern California. In addition to comprehensive exposure, dietary, and health history data, the study established a biorepository of blood, urine, and saliva samples. The long-term aims of the study are to (1) identify biomarkers, especially epigenetic markers, of environmental exposures, such as pesticides, air/food/water contaminants, and industrial chemicals that are commonly encountered in the general population; and (2) support the study of potential relationships between environmental exposures and health and health-related factors. While studies have been initiated to identify DNA methylation markers for some environmental exposures by the study investigators, the MEE Study is also intended to serve as a resource for researchers beyond the original investigators.

## Figures and Tables

**Table 1:** Data and specimens collected.

**Data Sources**	**Description**
Environmental exposures questionnaire	Environmental exposures, source of drinking water, organic eating behaviors, residence history, occupation
Breast health questionnaire	Personal medical history, reproductive history, family history of cancer, demographic data
Dietary recalls (ASA24)	Complete report of all food, drink, and supplements consumed the previous day (3 recalls were requested)
Food frequency questionnaire (DHQII)	Summary of dietary intake frequencies over the previous year
Electronic medical record	Mammogram reports
**Specimens Collected**	**Description**
Blood	Peripheral blood, separated into serum, plasma, and buffy coat; DNA extracted from buffy coat
Urine	Two first-void urine samples
Saliva	Optional saliva sample

**Table 2:** Cohort characteristics for the MEE Study.

Characteristic	Mean (SD) or *N* (%)
**Age, years, mean (SD)**	56.7 (4.6)
**Race/Ethnicity, *N* (%)**	
Non-Hispanic white	257 (64.3)
Hispanic	74 (18.5)
Asian	43 (10.8)
Other/Unknown	26 (6.5)
**Education, *N* (%)**	
High school graduate or less	34 (8.5)
Some college or technical school	87 (21.8)
College graduate or more	277 (69.3)
Missing	2 (0.5)
**Occupation, *N* (%), out of 250**	
Unemployed or disabled	11 (4.4)
Homemaker	12 (4.8)
Retired	23 (9.2)
Employed (Full-time or Part-time)	204 (81.6)
**Current residence, *N* (%)**	
Los Angeles County	27 (6.8)
North Orange County	185 (46.2)
South Orange County	178 (44.5)
Other	10 (2.6)
**Diet questionnaires completed, *N* (%)**	
ASA24 dietary recall	
None	21 (5.3)
1	33 (8.3)
2	76 (19.0)
≥3	270 (67.5)
Paired urine and ASA dietary recall *	
None	56 (14)
1	110 (27.5)
2	234 (58.5)
Food frequency questionnaire	263 (65.8)

* A dietary recall was considered to be “paired” with a urine sample if the recall was provided for the day immediately prior to the first-void urine sample.

**Table 3:** Lifestyle factors and health histories among study participants.

Characteristic	Mean (SD) or *N* (%)
**Smoking status, *N* (%)**	
Current smoker	17 (4.3)
Former smoker	90 (22.5)
Never-smoker	292 (73.0)
Missing	1 (0.3)
**Alcohol consumption, *N* (%)**	
Never	106 (26.5)
Less than 2 drinks per week	166 (41.5)
2–7 drinks per week	74 (18.5)
More than 7 per week	51 (12.8)
Missing	3 (0.8)
**Weekly physical activity meets the Physical Activity Guidelines for Americans, *N* (%)**	
No	226 (56.5)
Yes	156 (39.0)
Missing	18 (4.5)
**BMI, kg/m^2^, mean (SD)**	26.8 (6.5)
**Age of menarche, mean (SD)**	12.8 (1.5)
**Pregnancy history**	
Number of live births, *N* (%)	
0	87 (21.8)
1	74 (18.5)
2	141 (35.3)
3	74 (18.5)
More than 3	24 (6.0)
Age at first birth, mean (SD)	27.7 (6.2)
**Age of menopause, mean (SD)**	48.7 (6.1)
**History of gynecologic surgery, *N* (%)**	
Oophorectomy	84 (21.0)
Hysterectomy	99 (24.8)
**Hormone replacement therapy use, *N* (%)**	
Never	255 (63.8)
Previous	62 (15.5)
Current	82 (20.5)
Missing	1 (0.3)
**Mammographic breast density, *N* (%)**	
Almost entirely fatty	42 (10.5)
Scattered fibroglandular densities	110 (27.5)
Heterogeneously dense	163 (40.8)
Extremely dense	77 (19.3)
Missing	8 (2.0)
**Family history of cancer in first-degree relatives, *N* (%)**	
Breast cancer (invasive or ductal carcinoma in situ [DCIS])	83 (21.1)
Ovarian cancer	13 (3.3)

**Table 4:** Environmental factors among study participants.

Characteristic	Mean (SD) or *N* (%)
**Organic food consumption frequency, *N* (%)**	
Often or always	127 (31.8)
Sometimes	114 (28.5)
Seldom or never	158 (39.5)
Missing	1 (0.3)
**Source of drinking water, *N* (%)**	
Tap water (without filter)	36 (9.0)
Bottled water	158 (39.5)
Filtered water	204 (51.0)
Don’t know or not sure	1 (0.3)
Missing	1 (0.3)
**History of living on a farm, *N* (%)**	
> 10 years	22 (5.5)
≤ 10 years	32 (8.0)
None	346 (86.5)
**Age when started living on a farm, mean (SD)**	8.8 (11.2)
**Used pesticides at home or workplace within** **past 7 days, *N* (%), out of 250**	
No	174 (69.6)
Yes	50 (20.0)
Don’t know or not sure	26 (10.4)

## Supplementary Materials

The following are available online at https://www.mdpi.com/1660-4601/17/5/1774/s1, Table S1: Full Text of Study Questionnaires.
