# Supplementary material for: Rationale, Study Design, and Cohort Characteristics for the Markers for Environmental Exposures (MEE) Study
Source: Int J Environ Res Public Health. 2020 Mar 9;17(5):1774. doi: 10.3390/ijerph17051774 (PMC7084413; doi:10.3390/ijerph17051774)
Supplement: Supplementary file 1 [file ijerph-17-01774-s001.zip › ijerph-734011-SI.docx]

**Table S1.** Full text of study questionnaires.

| **Environmental Exposures Questionnaire** | | |
| --- | --- | --- |
| **ID** | **Question** | **Response Choices** |
| 1 | Do you eat organic food? ["Organic" food is food that is EITHER labeled "USDA Organic", purchased locally from an "organic farm", grown without pesticides in a home garden, or raised on organic feed without hormones and without antibiotics.] | Seldom or never  Sometimes  Often or always  Don't know or not sure |
| 2 | If you eat fresh fruit or drink fruit juice, how often is that fruit or fruit juice organic? | I do not eat the food  Seldom or never  Sometimes  Often or always  Don’t know or not sure |
| 3 | How long have you been eating/drinking organic fruit/fruit juice? | More than 10 years  5-10 years  1-5 years  Less than 1 year |
| 4 | If you eat fresh vegetables or drink vegetable juice, how often is that vegetable or vegetable juice organic? | I do not eat the food  Seldom or never  Sometimes  Often or always  Don’t know or not sure |
| 5 | How long have you been eating/drinking organic vegetables or vegetable juice? | More than 10 years  5-10 years  1-5 years  Less than 1 year |
| 6 | If you eat grains (for example, wheat, rice), how often are those grains organic? | I do not eat the food  Seldom or never  Sometimes  Often or always  Don’t know or not sure |
| 7 | How long have you been eating organic grains? | More than 10 years  5-10 years  1-5 years  Less than 1 year |
| 8 | If you eat meat, how often is that meat organic? | I do not eat the food  Seldom or never  Sometimes  Often or always  Don’t know or not sure |
| 9 | How long have you been eating organic meat? | More than 10 years  5-10 years  1-5 years  Less than 1 year |
| 10 | If you eat eggs, how often are those eggs organic? | I do not eat the food  Seldom or never  Sometimes  Often or always  Don’t know or not sure |
| 11 | How long have you been eating organic eggs? | More than 10 years  5-10 years  1-5 years  Less than 1 year |
| 12 | If you eat dairy products (for example, milk, cheese), how often are those dairy products organic? | I do not eat the food  Seldom or never  Sometimes  Often or always  Don’t know or not sure |
| 13 | How long have you been eating organic dairy products? | More than 10 years  5-10 years  1-5 years  Less than 1 year |
| 14 | How often do you eat a meal or snack from a place such as McDonald's, In-N-Out, Subway, Burger King, Chik Fil-A, Wendy's, Arby's, Taco Bell, Pizza Hut, or Kentucky Fried Chicken? | Never  1-5 times/year  6-11 times/year  1-3 times/month  1 time/week  2-3 times/week  4-6 times/week  1 time/day  2 or more times/day |
| 15 | Other than the types of eating establishments in the previous question, how often do you eat a meal or snack from other eating establishments? | Never  1-5 times/year  6-11 times/year  1-3 times/month  1 time/week  2-3 times/week  4-6 times/week  1 time/day  2 or more times/day |
| 16 | How often do you eat a meal or snack that has been prepared at a deli or grocery store? | Never  1-5 times/year  6-11 times/year  1-3 times/month  1 time/week  2-3 times/week  4-6 times/week  1 time/day  2 or more times/day |
| 17 | How often do you eat meals that have been home-cooked from basic, simple ingredients (excluding pre-made meals)? | 1-5 times/year  6-11 times/year  1-3 times/month  1 time/week  2-3 times/week  4-6 times/week  1 time/day  2 or more times/day |
| 18 | Have you ever lived on a farm? | Yes  No |
| 19 | For how many total years did you live on a farm? | Less than 1 year  1-5 years  6-10 years  More than 10 years |
| 20 | How old were you when you STARTED living on a farm? | Numeric |
| 21 | What is your primary source of drinking water? | Tap water  Bottled water  Other (please specify)  Don't know or not sure |
| 22 | (if "other") Please indicate your primary source of drinking water. | Free text |
| 23 | Is the tap water you use for drinking or cooking filtered or treated in the home to remove chemicals and minerals? (This includes filtering pitchers like Brita or PUR and filters in your refrigerator or faucet but does NOT include water softeners) | Yes  No  Don't know or not sure |
| 24 | Please list the ZIP code OR city and state OR country of the CURRENT place you live. | Free text |
| 25 | How many years have you lived at your CURRENT residence? | Numeric |
| 26 | Have you lived at a PREVIOUS place (for at least one year)? | Yes  No |
| 27 | Please list the ZIP code OR city and state OR country of the PREVIOUS place you lived. | Free text |
| 28 | How many years did you live at the past place listed directly above? | Numeric |
| 29 | Have you lived at another PREVIOUS place (for at least one year)? | Yes  No |
| 30 | Please list the ZIP code OR city and state OR country of the PREVIOUS place you lived. | Free text |
| 31 | How many years did you live at the past place listed directly above? | Numeric |
| 32 | Have you lived at another PREVIOUS place (for at least one year)? | Yes  No |
| 33 | Please list the ZIP code OR city and state OR country of the PREVIOUS place you lived. | Free text |
| 34 | How many years did you live at the past place listed directly above? | Numeric |
| 35 | Have you lived at another PREVIOUS place (for at least one year)? | Yes  No |
| 36 | Please list the ZIP code OR city and state OR country of the PREVIOUS place you lived. | Free text |
| 37 | How many years did you live at the past place listed directly above? | Numeric |
| 38 | What is your current job status? (Mark the one that best describes you. If more than one describes you, mark both.) | Not working  Retired  Homemaker, raising children/grandchildren, care of others  Employed (full-time or part-time)  Disabled, unable to work  Other (please specify) |
| 39 | (if "other") Please describe. | Free text |
| 40 | What is/was your occupation? | Free text |
| 41 | In the past 7 days, were any chemical products used in your home to control fleas, roaches, ants, termites, or other insects? | Yes  No  I don't know |
| 42 | What kind(s) of insecticides did you use (name of product or take a picture)? | Free text |
| 43 | In the past 7 days, were any chemical products used in your lawn or garden to kill weeds? | Yes  No  I don't know |
| 44 | What kind(s) of weedkiller(s) did you use (name of product or take a picture)? | Free text |
| 45 | In the past 7 days, did you use any chemical products to control fleas, roaches, ants, termites, or other insects, or weeds, AT WORK? | Yes  No  I don't know |
| 46 | Do you play golf? | Never  Occasionally  Once per week  More than once per week |
| 47 | Have you ever smoked regularly for 6 months or more? | Yes  No  Don't know |
| 48 | Now, do you smoke cigarettes every day, some days, or not at all? | Every day  Some days  Not at all |
| 49 | How old were you when you last smoked fairly regularly? | Numeric |
| 50 | During the years you smoked, how many cigarettes did you usually smoke per day? | Numeric |
| 51 | During the past 30 days, on the days that you did smoke, about how many cigarettes did you usually smoke per day? | Numeric |
| 52 | Do you have the tendency to flush (get red or pink) in the face immediately after drinking a glass of beer (~6 fluid ounces) or 2/3 glass of wine or a shot of hard liquor? | Yes  No  Don't know |
| 53 | During the first to second year after you started drinking alcohol, did you have a tendency to flush in the face immediately after drinking a glass of beer (~6 fluid ounces) or 2/3 glass of wine or a shot of hard liquor? | Yes  No  Don't know |
| 54 | Have you ever taken any of the following hormone therapies (female hormones prescribed for women after menopause)? (Please check all that apply) | Estrogen only (e.g., Premarin)  Progestin only (for example, Provera)  Estrogen and progestin combination (for example, Prempro)  Estrogen and testosterone combination (for example, Estratest)  Natural hormone therapy (Herbal supplements)  Topical vaginal estrogen cream  Estrogen patch  Other hormone therapy  Yes - But not sure what kind of hormone replacement therapy  No - I have never been on any hormone therapy |
| 55 | Are you CURRENTLY taking hormone replacement (female hormones prescribed for women after menopause)? (Please check all that apply) | Estrogen only (e.g., Premarin)  Progestin only (for example, Provera)  Estrogen and progestin combination (for example, Prempro)  Estrogen and testosterone combination (for example, Estratest)  Natural hormone therapy (Herbal supplements)  Topical vaginal estrogen cream  Estrogen patch  Other hormone therapy  Yes - But not sure what kind of hormone replacement therapy  No - I am not currently on any hormone therapy |
| 56 | For how many total years were you or have you been on hormone replacement therapy? | Less than 5 years  For five years or more  Don't know |
| 57 | Are you CURRENTLY taking any of the following medications? (Please check all that apply) | Tamoxifen (Nolvadex)  Raloxifene (Evista)  Anastrozole (Arimidex)  Hormones for birth control  None |
| 58 | How many years have you been taking Tamoxifen (Nolvadex)? | Numeric |
| 59 | How many years have you been taking Raloxifene (Evista)? | Numeric |
| 60 | How many years have you been taking Anastrozole (Arimidex)? | Numeric |
| 61 | How many years have you been taking hormones for birth control? | Numeric |
| 62 | What is your current weight? | Numeric |
| **Breast Health Questionnaire** | | |
| **ID** | **Question** | **Response Choices** |
| 1 | What is your gender? | Female  Male |
| 2 | Are you currently pregnant or possibly pregnant? | No  Yes  Don't know |
| 3 | Are you currently breast feeding? | No  Yes |
| 4 | When was your last mammogram? | Less than 1 year ago  1 to 2 years ago  2 to 3 years ago  4 or more years ago  I've never had a mammogram  Don't know |
| 5 | Please list medical facility, city and state where your last mammogram was done: | Free text |
| 6 | Have you had a clinical breast exam within the last 3 months (done by a doctor or health care provider)? | Yes  No  Don't know |
| 7 | Did your doctor or other health care provider discover a new or unusual lump? | Yes  No  Don't know |
| 8 | Have you noticed any of the following changes in your RIGHT breast in the LAST 3 MONTHS? [Choose all that apply] | Lump (new or unusual)  Nipple discharge  Pain  Other  No, I haven't noticed any changes |
| 9 | Have you noticed any of the following changes in your LEFT breast in the LAST 3 MONTHS? [Choose all that apply] | Lump (new or unusual)  Nipple discharge  Pain  Other  No, I haven't noticed any changes |
| 10 | Have you noticed any of the following changes in your RIGHT breast that are PRESENT TODAY? [Choose all that apply] | Lump (new or unusual)  Nipple discharge  Pain  Other  No, I haven't noticed any changes |
| 11 | Have you noticed any of the following changes in your LEFT breast that are PRESENT TODAY? [Choose all that apply] | Lump (new or unusual)  Nipple discharge  Pain  Other  No, I haven't noticed any changes |
| 12 | The reason for your scheduled mammogram is: | Routine checkup  Other |
| 13 | Has a doctor ever told you that you have breast cancer or DCIS (ductal carcinoma in situ)? [Choose all that apply] | Yes - invasive breast cancer  Yes - ductal carcinoma in situ (DCIS)  Yes - but I don't know what kind of breast cancer  No  Don't know |
| 14 | In which breast were you diagnosed with DCIS (ductal carcinoma in situ)? | Left  Right  Both  Don't know |
| 15 | How old were you when you were first diagnosed with DCIS (ductal carcinoma in situ)? | Numeric |
| 16 | How old were you when you were first diagnosed with DCIS (ductal carcinoma in situ) in your other breast? | Numeric |
| 17 | In which breast were you diagnosed with breast cancer? | Left  Right  Both  Don't know |
| 18 | How old were you when you were first diagnosed with breast cancer? | Numeric |
| 19 | How old were you when you were first diagnosed with breast cancer in your other breast? | Numeric |
| 20 | Considering your health over the last month, how would you characterize your health? | Excellent  Very Good  Good  Fair  Poor |
| 21 | Has a doctor ever told you that you have any of the following health conditions? [Choose all that apply] | Alzheimer's disease, dementia  Anemia or other blood disease  Back Pain  Being Overweight (Obesity)  Cancer (other than breast cancer)  Chronic Obstructive Pulmonary Disease  Congestive Heart Failure  Depression  Diabetes (problem with your blood sugar levels)  Heart Attack  Heart Disease  High Blood Pressure  Kidney Disease  Liver Disease  Lung Disease (e.g., asthma, pulmonary fibrosis, etc.)  Osteoarthritis, degenerative arthritis  Rheumatoid arthritis  Stroke  Thyroid Disease  Ulcer or Stomach Disease  Don't know  None  Other conditions (not listed) |
| 22 | Which type(s) of the following cancers have you had? [Choose all that apply] | Adrenal Cancer  Bladder Cancer  Bone Cancer  Brain Cancer  Breast Cancer  Cervical Cancer  Colon, Rectal, Large Intestine Cancer  Esophageal Cancer  Gall Bladder Cancer  Gastric/Stomach Cancer  Small Intestine Cancer  Kidney or Renal Cancer  Leukemia  Lung Cancer  Lymphoma - Hodgkins  Lymphoma - non-Hodgkins  Ovarian, Peritoneal, Fallopian Tube Cancer  Pancreatic Cancer  Prostate Cancer  Renal Pelvis / Ureter Cancer  Sarcoma  Skin - Basal / Squamous Cell Cancer  Skin - Melanoma Cancer  Testicular Cancer  Thyroid Cancer  Uterine (non-Cervical) / Endometrial Cancer  Unknown Type of Cancer  Other |
| 23 | When were you diagnosed with ovarian cancer? (Date can be estimated if exact date is not known) | Date |
| 24 | Have you ever had chemotherapy? | No  Yes  Don't know |
| 25 | How old were you when you had chemotherapy? | Numeric |
| 26 | Why did you have chemotherapy? | Free text |
| 27 | Have you ever had radiation therapy on your chest wall prior to the age of 35? | No  Yes  Don't know |
| 28 | How old were you when you received radiation therapy on your chest wall? | Numeric |
| 29 | Why did you have radiation therapy on your chest wall? | Free text |
| 30 | Are you adopted? | Yes  No |
|  |  | Don't know |
| 31 | Do you know the medical history from your biological family? | Yes  No  Don't know |
| *Instructions: Please answer the following questions for your blood relatives, including half-relatives and relatives who have died, but not step or adoptive relatives.* | | |
| 32 | How many sisters do you have? | Numeric |
| 33 | How many daughters do you have? | Numeric |
| 34 | How many maternal aunts (mom's sisters) do you have? | Numeric |
| 35 | How many paternal aunts (father's sisters) do you have? | Numeric |
| 36 | Which of your blood relatives have ever been diagnosed with breast cancer or ductal carcinoma in situ (DCIS)? [Choose all that apply] | Mother  Sister(s)  Daughter(s)  Maternal grandmother (mother's mother)  Paternal grandmother (father's mother)  Maternal aunt(s) - mother's sisters  Paternal aunt(s) - father's sisters  Any male relatives  Don't know  None of the above |
| 37 | Please specify if your mother was diagnosed with breast cancer or ductal carcinoma in situ (DCIS). [Choose all that apply] | Invasive Breast Cancer  Ductal carcinoma in situ (DCIS)  I don't know what kind of breast cancer |
| 38 | At about what age did your mother have breast cancer? (Please estimate if you are not sure) | Numeric |
| 39 | At about what age did your mother have DCIS (ductal carcinoma in situ)? (Please estimate if you are not sure) | Numeric |
| 40 | Did your mother have breast cancer or DCIS in BOTH breasts (bilateral)? | Yes  No  Don't know |
| 41 | Please specify if your sister(s) were diagnosed with breast cancer or ductal carcinoma in situ (DCIS). [Choose all that apply] | Invasive Breast Cancer  Ductal carcinoma in situ (DCIS)  I don't know what kind of breast cancer |
| 42 | How many sisters have ever been diagnosed with breast cancer? | Numeric |
| 43 | How many sisters have ever been diagnosed with DCIS (ductal carcinoma in situ)? | Numeric |
| 44 | At about what age did your sister(s) have breast cancer (if more than one sister had breast cancer, please select youngest age of diagnosis)? (Please estimate if you are not sure) | Numeric |
| 45 | At about what age did your sister(s) have DCIS (if more than one sister had DCIS, please select youngest age of diagnosis)? (Please estimate if you are not sure) | Numeric |
| 46 | Did any of your sisters have breast cancer or DCIS in BOTH breasts (bilateral)? | Yes  No  Don't know |
| 47 | Please specify if your daughter(s) were diagnosed with breast cancer or ductal carcinoma in situ (DCIS)? [Choose all that apply] | Invasive Breast Cancer  Ductal carcinoma in situ (DCIS)  I don't know what kind of breast cancer |
| 48 | How many of your daughters have ever been diagnosed with breast cancer? | Numeric |
| 49 | How many of your daughters have ever been diagnosed with DCIS (ductal carcinoma in situ)? | Numeric |
| 50 | At about what age did your daughter(s) have breast cancer (if more than one daughter had breast cancer, please select youngest age of diagnosis)? (Please estimate if you are not sure) | Numeric |
| 51 | At about what age did your daughter(s) have DCIS (if more than one daughter had DCIS, please select youngest age of diagnosis)? (Please estimate if you are not sure) | Numeric |
| 52 | Did any of your daughters have breast cancer or DCIS in BOTH breasts (bilateral)? | Yes  No  Don't know |
| 53 | Please specify if your maternal grandmother (mother's mother) was diagnosed with breast cancer or ductal carcinoma in situ (DCIS)? [Choose all that apply] | Invasive Breast Cancer  Ductal carcinoma in situ (DCIS)  I don't know what kind of breast cancer |
| 54 | At about what age did your maternal grandmother have breast cancer? (Please estimate if you are not sure) | Numeric |
| 55 | At about what age did your maternal grandmother have DCIS (ductal carcinoma in situ)? (Please estimate if you are not sure) | Numeric |
| 56 | Did your maternal grandmother have breast cancer or DCIS in BOTH breasts (bilateral)? | Yes  No  Don't know |
| 57 | Please specify if your paternal grandmother (father's mother) was diagnosed with breast cancer or ductal carcinoma in situ (DCIS)? [Choose all that apply] | Invasive Breast Cancer  Ductal carcinoma in situ (DCIS)  I don't know what kind of breast cancer |
| 58 | At about what age did your paternal grandmother have breast cancer? (Please estimate if you are not sure) | Numeric |
| 59 | At about what age did your paternal grandmother have DCIS (ductal carcinoma in situ)? (Please estimate if you are not sure) | Numeric |
| 60 | Did your paternal grandmother have breast cancer or DCIS in BOTH breasts (bilateral)? | Yes  No  Don't know |
| 61 | Please specify if your maternal aunt(s) (mother's sisters) were diagnosed with breast cancer or ductal carcinoma in situ (DCIS)? [Choose all that apply] | Invasive Breast Cancer  Ductal carcinoma in situ (DCIS)  I don't know what kind of breast cancer |
| 62 | How many of your maternal aunts (mother's sisters) have been diagnosed with breast cancer? | Numeric |
| 63 | How many of your maternal aunts (mother's sisters) have been diagnosed DCIS (ductal carcinoma in situ)? | Numeric |
| 64 | At about what age did your maternal aunt(s) have breast cancer (if more than one maternal aunt had breast cancer, select youngest age of diagnosis)? (Please estimate if you are not sure) | Numeric |
| 65 | At about what age did your maternal aunt(s) have DCIS (if more than one maternal aunt had DCIS, select youngest age of diagnosis)? (Please estimate if you are not sure) | Numeric |
| 66 | Did any of your maternal aunts have breast cancer or DCIS in BOTH breasts (bilateral)? | Yes  No  Don't know |
| 67 | Please specify if your paternal aunt(s) (father's sisters) were diagnosed with breast cancer or ductal carcinoma in situ (DCIS)? [Choose all that apply] | Invasive Breast Cancer  Ductal carcinoma in situ (DCIS)  I don't know what kind of breast cancer |
| 68 | How many of your paternal aunts (father's sisters) have ever been diagnosed with breast cancer? | Numeric |
| 69 | How many of your paternal aunts (father's sisters) have been diagnosed with DCIS (ductal carcinoma in situ)? | Numeric |
| 70 | At about what age did your paternal aunt(s) have breast cancer (if more than one paternal aunt had breast cancer, select youngest age of diagnosis)? (Please estimate if you are not sure) | Numeric |
| 71 | At about what age did your paternal aunt(s) have DCIS (if more than one paternal aunt had DCIS, select youngest age of diagnosis)? (Please estimate if you are not sure) | Numeric |
| 72 | Did any of your paternal aunts have breast cancer or DCIS in BOTH breasts (bilateral)? | Yes  No  Don't know |
| 73 | How many of your close, blood, female relatives (mother, sisters, daughters, grandmothers, aunts) have ever been diagnosed with ovarian cancer? | Numeric |
| 74 | Which of your blood relatives have ever been diagnosed with ovarian cancer? [Choose all that apply] | Mother  Sister(s)  Daughter(s)  Maternal grandmother (mother's mother)  Paternal grandmother (father's mother)  Maternal aunt(s) - mother's sisters  Paternal aunt(s) - father's sisters  Don't know  None of the above |
| 75 | Have any of your close, blood, female relatives (mother, sisters, daughters, grandmothers, or aunts) ever been diagnosed with BOTH breast and ovarian cancers? | Yes  No  Don't know |
| 76 | Please check all of the statements below that apply to you: [Choose all that apply] | I have 2 or more relatives on the same side of my family who have been diagnosed with uterus (endometrial) and/or colon cancer before age 55  I have a niece or cousin who was diagnosed with breast cancer (or ductal carcinoma in situ (DCIS) before age 55  I have a relative who was diagnosed with any type of cancer before age 20  I have a relative who was diagnosed with leukemia, brain cancer, or a sarcoma before age 45  Don't know  None of the above |
| 77 | Have you or your relatives ever had genetic testing for breast cancer risk? [Choose all that apply] | Yes - I have  Yes - my relatives have  No  Don't know |
| 78 | Were you positive for a gene mutation (e.g., BRCA1, BRCA2, gene variant, etc.): | Yes  No  Don't know |
| 79 | Do you know what type of gene mutation you have? [Choose all that apply] | BRCA1  BRCA2  SNPs  P53  PTEN  CDH1  CHEK2  Gene variant  Other  Don't know |
| 80 | Were any of your relatives positive for a gene mutation (e.g., BRCA1, BRCA2, gene variant, etc.): | Yes  No  Don't know |
| 81 | Do you know what type of gene mutation your relative(s) have: [Choose all that apply] | BRCA1  BRCA2  P53  PTEN  CDH1  SNPs  CHEK2  Gene variant  Other  Don't know |
| 82 | How many times have you been pregnant? | Numeric |
| 83 | Have you ever given birth? | Yes  No |
| 84 | How many live births? | Numeric |
| 85 | How old were you when your first child was born? | Numeric |
| 86 | How old were you when you had your first menstrual period (please estimate if you're not sure)? | Under age 10  10  11  12  13  14  15  16  Over 16  Don't know |
| 87 | Have your menstrual periods stopped permanently? | Yes - Periods stopped naturally (menopause)  Yes - But now have periods induced by hormones  Yes - Uterus removed by surgery  Yes - Both ovaries removed by surgery  Yes - Uterus AND both ovaries removed by surgery  Yes - Uterus AND one ovary removed by surgery  Yes - Due to radiation  Yes - Other reason  No - my menstrual periods have not stopped  Not sure - periods less frequent |
| 88 | How old were you when your periods stopped? | Numeric |
| 89 | Have you ever had a hysterectomy (a surgery to remove your uterus or womb)? | Yes  No  Don't know |
| 90 | At what age did you have your hysterectomy? | Numeric |
| 91 | Have you ever had one, both, or part of an ovary removed (oophorectomy)? | Yes - both ovaries were removed  Yes - only one ovary was removed  Yes - only part of one ovary was removed  Yes - but I don't know whether one or both ovaries were removed  No - I have never had an oophorectomy  Don't know |
| 92 | Were your ovaries removed at the same time? | Yes - same time  No - different times  Don't know |
| 93 | At what age did you first have an ovary or part of an ovary removed? | Numeric |
| 94 | Have you had any of the following breast procedures on your RIGHT breast? [Choose all that apply] | Fine Needle Aspiration (FNA)  Core biopsy  Surgical biopsy  Lumpectomy for cancer  Mastectomy  Radiation Therapy  Breast reconstruction  Breast reduction  Implants  None of the above  Don't know |
| 95 | When did you have your FIRST fine needle aspiration (FNA) on your RIGHT breast? (Date can be estimated if exact date is not known) | Date |
| 96 | When did you have your FIRST core biopsy on your RIGHT breast? (Date can be estimated if exact date is not known) | Date |
| 97 | When did you have your FIRST surgical biopsy on your RIGHT breast? (Date can be estimated if exact date is not known) | Date |
| 98 | When did you have a lumpectomy for cancer on your RIGHT breast? (Date can be estimated if exact date is not known) | Date |
| 99 | When did you have a mastectomy on your RIGHT breast? (Date can be estimated if exact date is not known) | Date |
| 100 | When did you COMPLETE radiation therapy on your RIGHT breast? (Date can be estimated if exact date is not known) | Date |
| 101 | When did you have breast reconstruction on your RIGHT breast? (Date can be estimated if exact date is not known) | Date |
| 102 | When did you have breast reduction on your RIGHT breast? (Date can be estimated if exact date is not known) | Date |
| 103 | When did you get breast implants in your RIGHT breast? (Date can be estimated if exact date is not known) | Date |
| 104 | Have you had any of the following breast procedures on your LEFT breast? [Choose all that apply] | Fine Needle Aspiration (FNA)  Core biopsy  Surgical biopsy  Lumpectomy for cancer  Mastectomy  Radiation Therapy  Breast reconstruction  Breast reduction  Implants  None of the above  Don't know |
| 105 | When did you have your FIRST fine needle aspiration (FNA) on your LEFT breast? (Date can be estimated if exact date is not known) | Date |
| 106 | When did you have your FIRST core biopsy on your LEFT breast? (Date can be estimated if exact date is not known) | Date |
| 107 | When did you have your FIRST surgical biopsy on your LEFT breast? (Date can be estimated if exact date is not known) | Date |
| 108 | When did you have a lumpectomy for cancer on your LEFT breast? (Date can be estimated if exact date is not known) | Date |
| 109 | When did you have a mastectomy on your LEFT breast? (Date can be estimated if exact date is not known) | Date |
| 110 | When did you COMPLETE radiation therapy on your LEFT breast? (Date can be estimated if exact date is not known) | Date |
| 111 | When did you have breast reconstruction on your LEFT breast? (Date can be estimated if exact date is not known) | Date |
| 112 | When did you have breast reduction on your LEFT breast? (Date can be estimated if exact date is not known) | Date |
| 113 | When did you get breast implants in your LEFT breast? (Date can be estimated if exact date is not known) | Date |
| 114 | How many breast biopsies (e.g., needle biopsies, surgeries) have you had in total? | 0  1  More than 1  Don't know |
| 115 | Have any of your breast biopsies (e.g., needle biopsies, surgeries) showed atypical ductal hyperplasia, sometimes called atypia or ADH? | Yes  No  Don't know |
| 116 | Have any of your breast biopsies (e.g., needle biopsies, surgeries) shown LCIS (lobular carcinoma in situ)? | No  Yes  Don't know |
| 117 | Have you had a cyst aspiration on your breast(s)? | Yes, don't know which side  Yes, on both breasts  Yes, on my left breast  Yes, on my right breast  No  Don't know |
| 118 | When did you have your FIRST cyst aspiration on your RIGHT breast? (Date can be estimated if exact date is not known) | Date |
| 119 | When did you have your FIRST cyst aspiration on your LEFT breast? (Date can be estimated if exact date is not known) | Date |
| 120 | When did you have your FIRST cyst aspiration? (Date can be estimated if exact date is not known) | Date |
| 121 | What is your height in feet (inches will be asked in the next question)? | 3 feet  4 feet  5 feet  6 feet  7 feet  8 feet |
| 122 | What is your current height in inches? | 0 inches  1 inch  2 inches  3 inches  4 inches  5 inches  6 inches  7 inches  8 inches  9 inches  10 inches  11 inches |
| 123 | How often do you have a drink containing alcohol? | Never  Once per month or under  2-4 times per MONTH  2-3 times per WEEK  4 or more times per WEEK |
| 124 | On a typical day that you have alcohol, how many drinks do you have? | 1 or 2  3 or 4  5 or 6  7 to 9  10 or more |
| *Instructions: The following questions ask about four types of exercise:*  *1) walking*  *2) strenuous or very hard exercise (you work up a sweat and your heart beats fast)*  *3) moderate exercise (not exhausting)*  *4) mild exercise* | | |
| 125 | Think about the WALKING you do outside the home. How often do you walk outside the home for more than 10 minutes without stopping? | Rarely or never  1 - 3 times each month  1 time each week  2 - 3 times each week  4 - 6 times each week  7 or more times each week |
| 126 | When you walk outside the home for more than 10 minutes without stopping, for how many minutes do you usually walk? | Less than 20 min  20 - 39 minutes  40 - 59 minutes  1 hour or more |
| 127 | What is your usual speed of walking? | Casual strolling or walking (less than 2 miles an hour)  Average or normal (2-3 miles an hour)  Fairly fast (3-4 miles an hour)  Very fast (more than 4 miles an hour)  Don't know |
| 128 | Think about the STRENUOUS OR VERY HARD EXERCISE you do (you work up a sweat and your heart beats fast); for example, aerobics, aerobic dancing, jogging, tennis, swimming laps). How many days per week do you do this type of exercise? | none  1 day each week  2 days each week  3 days each week  4 days each week  5 or more days each week |
| 129 | How long do you usually exercise like this at one time? | Less than 20 min  20 - 39 minutes  40 - 59 minutes  1 hour or more |
| 130 | Think about the MODERATE exercise you do (not exhausting; for example, biking outdoors, using an exercise machine (like a stationary bike or treadmill), calisthenics, easy swimming, popular or folk dancing. How many days per week do you do this type of exercise? | none  1 day each week  2 days each week  3 days each week  4 days each week  5 or more days each week |
| 131 | How long do you usually exercise like this at one time? | Less than 20 min  20 - 39 minutes  40 - 59 minutes  1 hour or more |
| 132 | Think about the MILD exercise you do; for example, slow dancing, bowling, golf, yoga. How many days per week do you do this type of exercise? | none  1 day each week  2 days each week  3 days each week  4 days each week  5 or more days each week |
| 133 | How long do you usually exercise like this at one time? | Less than 20 min  20 - 39 minutes  40 - 59 minutes  1 hour or more |
| 134 | What is your racial background? [Choose all that apply] | Black or African American  White  Asian  American Indian or Alaska Native  Native Hawaiian or Other Pacific Islander  Some other race  Don't know  Prefer not to answer |
| 135 | What is your ASIAN background? [Choose all that apply] | Chinese  Filipino  Asian Indian  Japanese  Korean  Vietnamese  Other Asian  Don't know |
| 136 | What is your PACIFIC ISLANDER background? [Choose all that apply] | Native Hawaiian  Samoan  Guamanian or Chamorro  Other Pacific Islander  Don't know |
| 137 | Are you of Hispanic, Latino or Spanish origin or ancestry? | No, not of Hispanic, Latino or Spanish origin  Yes - Mexican, Mexican American, or Chicano  Yes - Puerto Rican  Yes - Cuban  Yes - other Hispanic, Latino, or Spanish origin  Prefer not to answer |
| 138 | Do you have any Jewish ancestry in your family? | Yes - only on my father's side  Yes - only on my mother's side  Yes - on both sides  Yes - not sure which side of my family  No  Don't know  Prefer not to answer |
| 139 | What best describes your current marital status? | Married  Widowed  Living with a partner in a marriage-like relationship  Never married  Divorced  Separated |
| 140 | How many years of schooling have you had? | Some high school or less  High school graduate  Some college or technical school  College graduate or more |
